# Adenoma surveillance and colorectal cancer incidence: a retrospective, multicentre, cohort study

**DOI:** 10.1016/S1470-2045(17)30187-0

**Published:** 2017-06

**Authors:** Wendy Atkin, Kate Wooldrage, Amy Brenner, Jessica Martin, Urvi Shah, Sajith Perera, Fiona Lucas, Jeremy P Brown, Ines Kralj-Hans, Paul Greliak, Kevin Pack, Jill Wood, Ann Thomson, Andrew Veitch, Stephen W Duffy, Amanda J Cross

**Affiliations:** a Cancer Screening and Prevention Research Group, Department of Surgery and Cancer, Imperial College London, London, UK; b New Cross Hospital, Wolverhampton, UK; c Centre for Cancer Prevention, Wolfson Institute of Preventive Medicine, Queen Mary University, London, UK

## Abstract

**Background:**

Removal of adenomas reduces colorectal cancer incidence and mortality; however, the benefit of surveillance colonoscopy on colorectal cancer risk remains unclear. We examined heterogeneity in colorectal cancer incidence in intermediate-risk patients and the effect of surveillance on colorectal cancer incidence.

**Methods:**

We did this retrospective, multicentre, cohort study using routine lower gastrointestinal endoscopy and pathology data from patients who, after baseline colonoscopy and polypectomy, were diagnosed with intermediate-risk adenomas mostly (>99%) between Jan 1, 1990, and Dec 31, 2010, at 17 hospitals in the UK. These patients are currently offered surveillance colonoscopy at intervals of 3 years. Patients were followed up through to Dec 31, 2014.We assessed the effect of surveillance on colorectal cancer incidence using Cox regression with adjustment for patient, procedural, and polyp characteristics. We defined lower-risk and higher-risk subgroups on the basis of polyp and procedural characteristics identified as colorectal cancer risk factors. We estimated colorectal cancer incidence and standardised incidence ratios (SIRs) using as standard the general population of England in 2007. This trial is registered, number ISRCTN15213649.

**Findings:**

253 798 patients who underwent colonic endoscopy were identified, of whom 11 944 with intermediate-risk adenomas were included in this analysis. After a median follow-up of 7·9 years (IQR 5·6–11·1), 210 colorectal cancers were diagnosed. 5019 (42%) patients did not attend surveillance and 6925 (58%) attended one or more surveillance visits. Compared to no surveillance, one or two surveillance visits were associated with a significant reduction in colorectal cancer incidence rate (adjusted hazard ratio 0·57, 95% CI 0·40–0·80 for one visit; 0·51, 0·31–0·84 for two visits). Without surveillance, colorectal cancer incidence in patients with a suboptimal quality colonoscopy, proximal polyps, or a high-grade or large adenoma (≥20 mm) at baseline (8865 [74%] patients) was significantly higher than in the general population (SIR 1·30, 95% CI 1·06–1·57). By contrast, in patients without these features, colorectal cancer incidence was lower than that of the general population (SIR 0·51, 95% CI 0·29–0·84).

**Interpretation:**

Colonoscopy surveillance benefits most patients with intermediate-risk adenomas. However, some patients are already at low risk after baseline colonoscopy and the value of surveillance for them is unclear.

**Funding:**

National Institute for Health Research Health Technology Assessment, Cancer Research UK.

## Introduction

Colorectal cancer is a major cause of cancer morbidity and death in developed countries.^1^ Endoscopic removal of adenomas, precursors of most colorectal cancers, reduces colorectal cancer incidence and mortality.^2^, ^3^, ^4^, ^5^ Patients perceived to be at increased risk after adenoma removal are recommended surveillance colonoscopy.^6^, ^7^, ^8^, ^9^, ^10^

National guidelines for adenoma surveillance stratify patients into risk groups based mainly on the detection of advanced neoplasia (adenomas ≥10 mm or with advanced pathology, or cancer) in those attending follow-up colonoscopy as a surrogate for long-term colorectal cancer incidence. The risk of advanced neoplasia at follow-up colonoscopy depends on the number, size, and histology of baseline adenomas,^11^, ^12^, ^13^, ^14^ as well as the quality of the baseline examination.^15^, ^16^ UK, European Union (EU), and US guidelines define a low-risk group for which no surveillance, or surveillance at intervals of 5–10 years, is recommended, an intermediate-risk or higher-risk group for which surveillance every 3 years is recommended, and a high-risk group for which an additional clearing colonoscopy within either 12 months (UK and EU) or within 3 years (USA) is recommended before continuation with surveillance every 3 years.^6^, ^7^, ^8^, ^9^, ^10^ The recommendation for 3-yearly surveillance is based on the results of a randomised trial that showed that the cumulative advanced neoplasia detection rate was similar between patients who had one or two surveillance colonoscopies within 3 years.^17^ Although UK and US criteria for 3-yearly surveillance differ slightly (UK criteria are one-to-two adenomas ≥10 mm or three-to-four adenomas <10 mm, whereas US criteria are 3–10 adenomas or any adenomas ≥10 mm, with villous architecture or high-grade dysplasia), advanced neoplasia detection rates at follow-up colonoscopy are similar, at 10% in the UK versus 11% in the USA.^18^ The UK guideline recommends stopping 3-yearly surveillance after two consecutive negative colonoscopies (appendix p 1), whereas in the USA, there are no recommended criteria for stopping other than older age.

**Research in context**

**Evidence before this study**

Before the start of the study in 2006, we searched MEDLINE via PubMed for available evidence, although we did not complete a systematic review. The existing guidelines for colonoscopic surveillance after adenoma detection were developed in 2002. High-risk, intermediate-risk, and low-risk groups were identified, and an appropriate surveillance strategy was developed for each. This guideline was accepted by the British Society of Gastroenterology and the National Institute for Health and Care Excellence. A 3 year surveillance interval was indicated for those at intermediate risk on the basis of evidence from a randomised trial that compared different surveillance intervals for the detection of advanced adenomas at follow-up. In 2012, a study in France investigated colorectal cancer risk in patients diagnosed with adenomas in the 1990s. The results of the study showed a clear benefit from surveillance in patients with one or more advanced adenomas, whereas in those with only non-advanced adenomas, the benefit was less marked. Nevertheless, the investigators concluded that gastroenterologists should encourage patients to comply with long-term surveillance. The study did not account for the confounding effects of colonoscopy quality on subsequent colorectal cancer risk. Evidence suggests that the quality of colonoscopy has improved and that the number of missed or incompletely removed lesions has decreased since the publication of a UK national colonoscopy audit in 2001, leading to implementation of national training standards and quality assessments. No study has yet assessed the effect of surveillance on long-term colorectal cancer risk among patients offered 3-yearly surveillance, who represent most patients offered surveillance.

**Added value of this study**

Our study assessed colorectal cancer risk in patients considered to be at intermediate risk. Across 8 years of follow-up, our data identified risk factors for colorectal cancer at baseline colonoscopy that permitted further stratification of these patients into lower-risk and higher-risk subgroups. Patients with an incomplete colonoscopy, poor bowel preparation, proximal polyps, or a high-grade or large adenoma (≥20 mm) at baseline were at increased risk, and the first surveillance colonoscopy significantly reduced colorectal cancer risk. By contrast, in patients without these baseline colonoscopy findings, future risk of colorectal cancer was already lower than that in the general population before any surveillance.

**Implications of all the available evidence**

Our results show that most patients who are currently offered 3-yearly surveillance colonoscopy benefit substantially from attending at least one surveillance visit. However, about a third of these patients are at low risk compared with the general population and are unlikely to benefit substantially from colonoscopy surveillance. About 20% of colonoscopies in the UK and 25% in the US are done for adenoma surveillance, which puts a huge pressure on endoscopy resources. Evidence from this study will be important in informing future adenoma surveillance guidelines and will help to minimise the costs and risks associated with unnecessary colonoscopies.

The main aim of adenoma surveillance is to reduce the incidence of colorectal cancer, but very few studies have used long-term colorectal cancer incidence after adenoma removal to define risk groups and need for surveillance^12^, ^19^, ^20^ and none have looked at predictive factors for long-term colorectal cancer incidence in patients who are currently offered surveillance. About 20% of colonoscopies in the UK and 25% in the USA are for adenoma surveillance,^21^, ^22^ which puts huge pressure on endoscopy resources. Any evidence that could help to minimise unnecessary colonoscopies while ensuring that colonoscopy surveillance is directed at patients at highest risk would be of timely importance. In this study, we estimated colorectal cancer incidence after baseline colonoscopy in patients who are recommended 3-yearly surveillance, and assessed the effect of surveillance on colorectal cancer incidence. We hypothesised that a subgroup of patients exists in whom surveillance colonoscopy could be stopped earlier, or for whom surveillance is not necessary, on the basis of their colorectal cancer incidence.

## Methods

### Study design and participants

We did this retrospective, multicentre, cohort study using information from 17 UK hospitals with electronic records of lower gastrointestinal endoscopy and pathology data recorded for at least 6 years before the start of the study in 2006 (appendix p 2). The size of the catchment population for the 17 hospitals was estimated to be more than 6·5 million people.^23^, ^24^

Patients were eligible for inclusion in the study if they had a baseline colonoscopy and newly diagnosed intermediate-risk adenomas according to UK guidelines, defined as one-to-two large (≥10 mm) adenomas, or three-to-four small adenomas (appendix p 1). We excluded patients with a history of bowel resection, colorectal cancer, inflammatory bowel disease, a family history of colorectal cancer, or any endoscopies without a date.

We searched gastrointestinal endoscopy databases to identify patients who underwent colonic examination before Dec 31, 2010, then we searched pathology databases for reports of colorectal lesions, using Systematised Nomenclature of Medicine (SNOMED) codes (versions 2 and 3), Systematized Nomenclature of Pathology (SNOP) codes, keywords, or multiple search terms. Endoscopy and pathology reports were linked and pseudonymised before being entered into an Oracle (11*g* Enterprise Edition) database. We coded patient, procedural, and polyp data using data entry constraints, standard operating procedures, and regular data audits to check coding consistency. Further details on hospital data collection and standard operating procedures are available in the appendices of our National Institute for Health Research (NIHR) Health Technology Assessment (HTA) report.^25^

We divided endoscopic examinations into visits (ie, one or more examinations made in close succession to complete a full examination of the colon and remove detected lesions). If there was evidence that a lesion had been incompletely removed, and a surveillance examination was scheduled soon after, we included that examination in the baseline visit. We used a hierarchy of rules to assign a summary value for the size, histology, and location of lesions seen at multiple examinations.^25^ Completeness of colonoscopy, and quality of bowel preparation^26^ were defined by the most complete examination and the best bowel preparation during the baseline visit. Baseline colonoscopy was defined as suboptimal if the most complete examination was incomplete or of unknown completeness or if the best bowel preparation was poor. Bowel preparation quality and completeness of colonoscopy, as assessed by the endoscopist, were obtained from endoscopy reports when not included as a separate field in the endoscopy database.

Patient, procedural, and polyp characteristics at baseline assessed as a-priori risk factors and confounders included age at first adenoma detection, sex, completeness of colonoscopy, quality of bowel preparation (graded as excellent, good, adequate or satisfactory, and poor),^26^ year of entry (year first adenoma detected), and adenoma number (total number recorded at baseline), size (largest at baseline), histology and grade of dysplasia (worst at baseline), and polyp location. We defined polyps as proximal if they were proximal to the descending colon. Data on lifestyle factors, such as smoking and alcohol consumption, were not available.

We ascertained the presence of colorectal cancers from hospital pathology reports and from National Health Service (NHS) Digital, the NHS Central Register (NHSCR), and National Services Scotland (NSS). Mortality data were provided by NHS Digital, NHSCR, and NSS.

Ethics approval was granted by the Royal Free Research Ethics Committee (reference 06/Q0501/45). Approval for use of patient information without consent was granted by the Patient Information Advisory Group under Section 60 of the Health and Social Care Act 2001 (PIAG 1–05[e]/2006). The study protocol is available online.

### Statistical analysis

The primary outcome was incident adenocarcinoma of the colorectum. This outcome excluded in-situ cancer. We assumed that cancers later diagnosed in lesions identified at baseline had been incompletely resected if baseline examinations showed that they were left intact or partly removed, were in the same or adjacent segment of the colon, and had similar histology; such cancers were excluded from the analysis.

Our sample size calculations stipulated that estimates of the colorectal cancer incidence rate have a coefficient of variation of about 30% (ie, the standard error of the estimate would be 30% of the actual estimate). Assuming conservatively a rate of two colorectal cancers per 1000 person-years,^20^, ^27^, ^28^ an approximate Poisson distribution of incidence, and a simple univariate estimate of the rate, then nine colorectal cancer events and 4500 person-years in any given subgroup would give a coefficient of variation of 33%. Assuming a smallest subgroup of interest of 15% of the cohort, we required at least 30 000 person-years (4500 divided by 0·15) and 60 colorectal cancers, or a total cohort of 6000 patients with at least 5 years of follow-up. Because inclusion of covariates might increase standard errors, we aimed to include at least 10 000 patients.

We censored time-to-event data at first colorectal cancer diagnosis, death, emigration, or December 31, 2014, for patients matched to national data sources or date of last recorded procedure for unmatched patients. Patients who could not be traced through national sources and who did not attend surveillance were excluded from the analysis. Time at risk started from the last examination at baseline, and exposure to successive surveillance visits started at the last procedure in each visit. Some analyses divided each patient's follow-up time into three distinct periods; without surveillance (from start of time at risk, censored at any first surveillance); after first surveillance (from first surveillance, censored at any second surveillance); and after second surveillance (from second surveillance to final date of censoring).

We compared baseline characteristics in patients with and without surveillance visits using χ^2^ tests. We created an unknown category for variables with missing data. We did not use multiple imputation or inverse probability weighting to deal with missing data.

We used one minus the Kaplan-Meier estimator of the survival function to show time to cancer diagnosis and to estimate the cumulative incidence of cancer with 95% CIs at 3, 5, and 10 years; we used the log-rank test to compare subgroups. We examined the effects of surveillance and patient, procedural, and polyp characteristics at baseline on long-term colorectal cancer incidence using Cox proportional hazards models.

We used univariable models to estimate unadjusted hazard ratios (HR) and 95% CIs. We identified independent predictors of colorectal cancer incidence in a multivariable model, using backward stepwise selection with a p value less than 0·05 in the likelihood ratio test as the criterion for retention of variables. The number of surveillance visits was included as a time-varying covariate and was constrained to be included in the multivariable model.

Using baseline polyp and procedural risk factors identified from the multivariable model, we stratified the intermediate-risk cohort into lower-risk and higher-risk subgroups. We did not include age as a factor in defining the higher-risk subgroup in our study because risks of adverse events increase with age coincidental with general decline in health, and older age is associated with worse colonoscopy quality.^29^, ^30^ We calculated expected numbers of colorectal cancers by multiplying the observed sex and 5-year age-group-specific person-years by the corresponding incidence in the general population of England in 2007.^31^ We report the ratio of observed to expected cases as a standardised incidence ratio (SIR) and 95% CIs assumed an exact Poisson distribution.

We did all analyses with Stata/IC 13.1. This study is registered with ISRCTN, number ISRCTN15213649.

### Role of the funding source

The funders of the study had no role in the study design, data collection, data analysis, data interpretation, or the writing of the report. KW, US, and WA had full access to all the data and WA had final responsibility for the decision to submit for publication.

## Results

**Figure 1:**
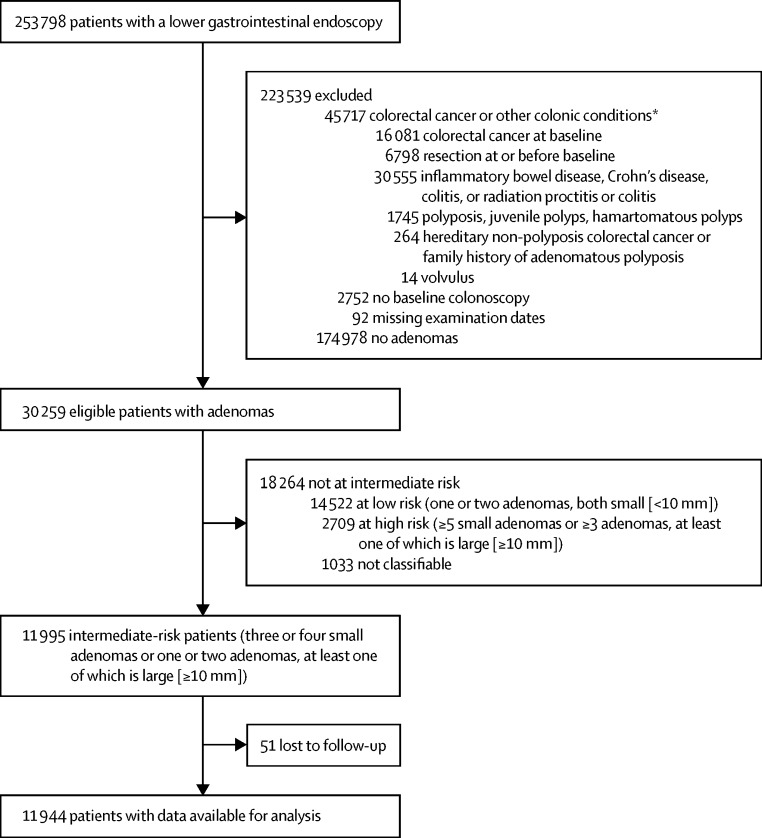
Study profile *Not mutually exclusive.

We identified 253 798 consecutive patients who underwent lower gastrointestinal endoscopies mostly (>99%) between Jan 1, 1990, and Dec 31, 2010. We excluded 223 539 patients: 174 978 with no adenomas, 45 717 with colorectal cancer or other conditions associated with increased colorectal cancer risk, 2752 with no colonoscopy, and 92 with missing procedure dates. Of the remaining 30 259 patients with a histologically confirmed adenoma at baseline, 11 995 (40%) were diagnosed with intermediate-risk adenomas, of whom 51 could not be traced in national data sources and had no surveillance, leaving 11 944 patients for analysis (figure 1).

The median age of 11 944 intermediate-risk patients was 66·7 years (IQR 58·4–74·0) and 6625 (55%) were men (table 1). The baseline visit included the first endoscopy at which an adenoma was observed and in most cases consisted of just one (6826 [57%] patients) or two (3788 [32%]) procedures; 19 072 (99·5%) of 19 164 baseline procedures were endoscopies and 92 (0·5%) were surgeries.

**Table 1:** Baseline patient, procedural, and polyp characteristics by surveillance visit attendance

		**Patients with one or more surveillance visits (n=6925)**	**Patients with no surveillance visits (n=5019)**	**p value**
Sex		..	..	0·0140
	Women	3018 (44%)	2301 (46%)	..
	Men	3907 (56%)	2718 (54%)	..
Age at first adenoma detection (years)		..	..	<0·0001
	<55	1490 (22%)	632 (13%)	..
	55–64	2205 (32%)	974 (19%)	..
	65–74	2370 (34%)	1587 (32%)	..
	≥75	860 (12%)	1826 (36%)	..
Number of adenomas		..	..	0·90
	1	4535 (65%)	3307 (66%)	..
	2	1789 (26%)	1284 (26%)	..
	3 or 4	601 (9%)	428 (9%)	..
Adenoma size (mm)		..	..	0·0002
	<10	601 (9%)	428 (9%)	..
	10–19	3869 (56%)	2988 (60%)	..
	≥20	2455 (35%)	1603 (32%)	..
Adenoma histology		..	..	<0·0001
	Tubular	2697 (39%)	2045 (41%)	..
	Tubulovillous	3284 (47%)	2292 (46%)	..
	Villous	623 (9%)	519 (10%)	..
	Unknown	321 (5%)	163 (3%)	..
Adenoma dysplasia		..	..	<0·0001
	Low-grade	5391 (78%)	4085 (81%)	..
	High-grade	1199 (17%)	795 (15%)	..
	Unknown	335 (5%)	139 (3%)	..
Proximal polyps		..	..	0·96
	No	4808 (69%)	3487 (69%)	..
	Yes	2117 (31%)	1532 (31%)	..
Completeness of colonoscopy		..	..	<0·0001
	Complete	5121 (74%)	3895 (78%)	..
	Incomplete	578 (8%)	749 (15%)	..
	Unknown	1226 (18%)	375 (7%)	..
Bowel preparation quality		..	..	<0·0001
	Excellent or good	2222 (32%)	1734 (35%)	..
	Satisfactory	906 (13%)	1016 (20%)	..
	Poor	270 (4%)	401 (8%)	..
	Unknown	3527 (51%)	1868 (37%)	..
Year of entry (start of baseline)		..	..	<0·0001
	1984–89	97 (1%)	15 (0%)	
	1990–94	233 (3%)	94 (2%)	..
	1995–99	1005 (15%)	425 (8%)	..
	2000–04	2542 (37%)	1709 (34%)	..
	2005–10	3048 (44%)	2776 (55%)	..

Data are n (%). p values calculated with χ^2^ test to compare patients with and without surveillance visits.

Compared with the 5019 (42%) patients who did not attend surveillance, the 6925 (58%) who attended one or more surveillance visits were younger, a greater proportion were male, and a greater proportion had a large adenoma (≥20 mm), an adenoma with high-grade dysplasia, an earlier date of diagnosis, and missing data. Although the p value for adenoma histology was significant there was no clear trend of increasing villousness between the groups. A lower proportion of patients who attended at least one surveillance visit had an incomplete colonoscopy or poor bowel preparation than did patients who did not attend any visits (table 1). The median time from baseline to first attended surveillance visit was 2·9 years (IQR 1·3–3·4).

During 101 034 person-years of follow-up (median 7·9 years, IQR 5·6–11·1), 3781 (32%) patients died and 210 colorectal cancers were diagnosed, giving an incidence rate of 208 events per 100 000 person-years (95% CI 182–238; table 2). Of the 5019 patients who did not attend surveillance, 2326 (46%) died and 121 (2%) were diagnosed with cancer, whereas of the 6925 patients who attended one or more surveillance visits, 1455 (21%) died and 89 (1%) were diagnosed with cancer. After adjustment for baseline risk factors, compared with no surveillance, one or two surveillance visits were associated with a significant reduction in colorectal cancer incidence rate (HR 0·57, 95% CI 0·40–0·80 for one visit; 0·51, 0·31–0·84 for two visits); a similar reduction in incidence rate was seen with three or more surveillance examinations (HR 0·54, 95% CI 0·29–0·99; table 2).

**Table 2:** Long-term colorectal cancer incidence by baseline risk factors and number of surveillance visits

		**n (%)**	**Person-years**	**Colorectal cancer cases**	**Incidence per 100 000 person-years (95% CI)**	**Univariable HR (95% CI)**	**p value**	**Multivariable HR (95% CI)**	**p value**
Total		11 944 (100%)	101 034	210	208 (182–238)	..	..	..	..
Number of surveillance visits after baseline*		..	..	..	..	..	0·0004	..	0·0029
	0	5019 (42%)	51 942	121	233 (195–278)	1	..	1	..
	1	3503 (29%)	29 503	51	173 (131–227)	0·54 (0·39–0·77)	..	0·57 (0·40–0·80)	..
	2	2085 (17%)	12 663	22	174 (114–264)	0·46 (0·28–0·75)	..	0·51 (0·31–0·84)	..
	≥3	1337 (11%)	6926	16	231 (142–377)	0·49 (0·27–0·88)	..	0·54 (0·29–0·99)	..
Sex		..	..	..	..	..	0·91	..	0·35
	Women	5319 (45%)	46 380	96	207 (169–253)	1	..	1	..
	Men	6625 (55%)	54 654	114	209 (174–251)	1·02 (0·77–1·33)	..	1·14 (0·86–1·50)	..
Age at first adenoma detection (years)		..	..	..	..	..	<0·0001	..	<0·0001
	<55	2122 (18%)	22 536	23	102 (68–154)	1	..	1	..
	55–64	3179 (27%)	30 039	39	130 (95–178)	1·33 (0·79–2·23)	..	1·28 (0·77–2·15)	..
	65–74	3957 (33%)	32 156	84	261 (211–324)	2·87 (1·80–4·57)	..	2·66 (1·66–4·24)	..
	≥75	2686 (22%)	16 304	64	393 (307–502)	4·72 (2·90–7·67)	..	3·82 (2·33–6·27)	..
Number of adenomas		..	..	..	..	..	0·12	NA†	NA†
	1	7842 (66%)	67 897	143	211 (179–248)	1	..	..	..
	2	3073 (26%)	24 785	57	230 (177–298)	1·12 (0·82–1·52)	..	..	..
	3 or 4	1029 (9%)	8353	10	120 (64–223)	0·58 (0·31–1·11)	..	..	..
Adenoma size (mm)		..	..	..	..	..	0·0495	..	0·0335
	<10	1029 (9%)	8353	10	120 (64–223)	1	..	1	..
	10–19	6857 (57%)	58 555	116	198 (165–238)	1·62 (0·85–3·09)	..	1·97 (1·01–3·81)	..
	≥20	4058 (34%)	34 126	84	246 (199–305)	2·02 (1·05–3·89)	..	2·28 (1·16–4·50)	..
Adenoma histology		..	..	..	..	..	0·0018	..	0·0348
	Tubular	4742 (40%)	40 404	64	158 (124–202)	1	..	1	..
	Tubulovillous	5576 (47%)	46 222	99	214 (176–261)	1·36 (1·00–1·87)	..	1·16 (0·84–1·61)	..
	Villous	1142 (10%)	9234	24	260 (174–388)	1·65 (1·03–2·64)	..	1·16 (0·71–1·91)	..
	Unknown	484 (4%)	5174	23	445 (295–669)	2·61 (1·61–4·23)	..	2·50 (1·40–4·47)	..
Adenoma dysplasia		..	..	..	..	..	0·0005	..	0·0033
	Low-grade	9476 (79%)	79 243	139	175 (149–207)	1	..	1	..
	High-grade	1994 (17%)	15 849	51	322 (245–423)	1·85 (1·34–2·55)	..	1·69 (1·21–2·36)	..
	Unknown	474 (4%)	5942	20	337 (217–522)	1·71 (1·06–2·77)	..	1·69 (1·04–2·76)	..
Proximal polyps		..	..	..	..	..	0·0285	..	0·0004
	No	8295 (69%)	72 301	137	189 (160–224)	1	..	1	
	Yes	3649 (31%)	28 733	73	254 (202–320)	1·38 (1·04–1·84)	..	1·76 (1·30–2·38)	
Completeness of colonoscopy		..	..	..	..	..	0·0007	..	0·0001
	Complete	9016 (75%)	72249	124	172 (144–205)	1	..	1	..
	Incomplete or not known	2928 (25%)	28785	86	299 (242–369)	1·64 (1·24–2·16)	..	1·80 (1·34–2·41)	..
Bowel preparation quality		..	..	..	..	..	0·0299	..	0·0452
	Excellent or good	3956 (33%)	33368	53	159 (121–208)	1		1	..
	Satisfactory	1922 (16%)	13609	29	213 (148–307)	1·41 (0·90–2·22)		1·51 (0·95–2·39)	..
	Poor	671 (6%)	4490	16	356 (218–582)	2·32 (1·33–4·06)		2·09 (1·19–3·67)	..
	Unknown	5395 (45%)	49567	112	226 (188–272)	1·37 (0·99–1·91)		1·39 (1·00–1·94)	..
Year of entry (start of baseline)		..	..	..	..	..	0·0562	..	0·0682
	1984–94	439 (4%)	6737	23	341 (227–514)	1	..	1	..
	1995–99	1430 (12%)	17390	51	293 (223–386)	0·86 (0·51–1·44)	..	0·86 (0·51–1·48)	..
	2000–04	4251 (36%)	39819	76	191 (152–239)	0·60 (0·36–0·99)	..	0·57 (0·33–0·99)	..
	2005–10	5824 (49%)	37088	60	162 (126–208)	0·54 (0·32–0·93)	..	0·55 (0·30–0·99)	..

The final multivariable model included number of surveillance visits, age, adenoma size, adenoma dysplasia, proximal polyps, completeness of colonoscopy, and bowel preparation quality; for these variables the multivariable HR reported was that from the final multivariable model and the p value was that for inclusion of the variable in the model from the likelihood ratio test. The multivariable HR and associated p value reported for sex, adenoma histology, and year of entry (variables not included in the final multivariable model), were for if the variable was added as an additional variable to the final multivariable model. Adenoma histology was not included in the final multivariable model because it was selected for inclusion only if the unknown category was included. HR=hazard ratio.

* Number of surveillance visits was included in the models as a time-varying covariate; if a patient had any surveillance visits, they contributed person-years to more than one category of number of surveillance visits.

† No multivariable hazard ratio and p value was reported for number of adenomas because of multicollinearity with largest adenoma size (largest size <10 mm perfectly predicts ≥3 adenomas).

Baseline characteristics independently associated with increased colorectal cancer incidence included older age, adenomas of 20 mm or larger, adenomas with high-grade dysplasia, polyps in the proximal colon, a colonoscopy that was incomplete or of unknown completeness, and poor quality bowel preparation (table 2). Adenoma histology was only significantly associated with colorectal cancer incidence if the unknown histology category was included in the model. Sex, number of adenomas, and year of entry were not independently associated with colorectal cancer incidence (table 2). Other baseline variables not included in the multivariable model are listed in the appendix (p 3).

On the basis of the polyp and procedural characteristics identified as colorectal cancer risk factors (but not older age), we divided the cohort into lower-risk (3079 [26%] patients) and higher-risk (8865 [74%]) subgroups. The higher-risk subgroup consisted of patients who, at baseline, had a large adenoma (≥20 mm), high-grade dysplasia, proximal polyps, or a suboptimal colonoscopy. The lower-risk subgroup consisted of patients without any of these findings. Colorectal cancer incidence was 247 cancers per 100 000 person-years (95% CI 214–285) in the higher-risk subgroup versus 93 cancers per 100 000 person-years (95% CI 63–139) in the lower-risk subgroup (table 3).

**Table 3:** Incidence of colorectal cancer and unadjusted effect of surveillance on incidence of colorectal cancer by number of visits

			**n (%)**	Person-years*	**Colorectal cancer cases**	**Incidence per 100 000 person-years (95% CI)**	Effect of surveillance*	
							Univariable HR (95% CI)	p value
Whole cohort			..	..	..	..	..	0·0001
	0 visits		5019 (42%)	51 942	121	233 (195–278)	1	..
	1 visit		3503 (29%)	29 503	51	173 (131–227)	0·54 (0·39–0·77)	..
	≥2 visits		3422 (29%)	19 589	38	194 (141–267)	0·47 (0·31–0·72)	..
	Total		11 944	101 034	210	208 (182–238)	..	..
Lower-risk subgroup†			..	..	..	..	..	0·22
	0 visits		1411 (46%)	14 861	15	101 (61–167)	1	..
	1 visit		937 (30%)	7095	6	85 (38–188)	0·54 (0·20–1·43)	..
	≥2 visits		731 (24%)	3749	3	80 (26–248)	0·36 (0·09–1·41)	..
	Total		3079 (26%)	25 705	24	93 (63–139)	..	..
Higher-risk subgroup†			..	..	..	..	..	0·0001
	0 visits		3608 (41%)	37 081	106	286 (236–346)	1	..
	1 visit		2566 (29%)	22 408	45	201 (150–269)	0·52 (0·36–0·75)	..
	≥2 visits		2691 (30%)	15 840	35	221 (159–308)	0·45 (0·29–0·70)	..
	Total		8865 (74%)	75 329	186	247 (214–285)	..	..
Reason classified as higher risk								
	Suboptimal quality examination only		..	..	..	..	..	0·25
		0 visits	613 (39%)	7121	18	253 (159–401)	1	..
		1 visit	451 (29%)	4720	8	170 (85–339)	0·49 (0·21–1·18)	..
		≥2 visits	490 (32%)	3491	10	286 (154–532)	0·81 (0·32–2·04)	..
		Total	1554 (13%)	15 331	36	235 (169–326)	..	..
	High-risk polyps only		..	..	..	..	..	0·0098
		0 visits	2223 (41%)	22 525	52	231 (176–303)	1	..
		1 visit	1631 (30%)	12 866	24	187 (125–278)	0·59 (0·36–0·98)	..
		≥2 visits	1620 (30%)	8083	14	173 (103–292)	0·40 (0·21–0·77)	..
		Total	5474 (46%)	43 475	90	207 (168–255)	..	..
	Both suboptimal quality examination and high-risk polyps		..	..	..	..	..	0·0084
		0 visits	772 (42%)	7434	36	484 (349–671)	1	..
		1 visit	484 (26%)	4822	13	270 (157–464)	0·44 (0·23–0·86)	..
		≥2 visits	581 (32%)	4267	11	258 (143–465)	0·34 (0·15–0·76)	..
		Total	1837 (15%)	16 524	60	363 (282–468)	..	..

p values calculated with the likelihood ratio test. HR=hazard ratio.

* Number of surveillance visits was included in the models as a time-varying covariate; if a patient had any surveillance visits, they contributed person-years to more than one category of number of surveillance visits.

† The higher-risk subgroup included patients with any of the following risk factors at baseline: suboptimal quality examination (defined as incomplete colonoscopy, unknown completeness, or poor bowel preparation), high-risk polyps (defined as proximal polyps or a high-grade or large [20mm or larger] adenoma), or both; the lower-risk subgroup included patients without any of these risk factors.

Patients in the higher-risk subgroup were older, had entered the study earlier, and had significantly more surveillance visits than those in the lower-risk subgroup (appendix p 4). However, median follow-up times were similar (8·0 years [IQR 5·5–11·3] in the higher-risk subgroup *vs* 7·8 years [5·7–10·6] in the lower-risk subgroup). Among higher-risk patients, number of surveillance visits was inversely associated with colorectal cancer incidence; by contrast, in the lower-risk subgroup, the number of surveillance visits was not associated with colorectal cancer incidence; however, statistical power was limited because of the low number of cancers (n=24 in total; table 3). In higher-risk patients with a suboptimal quality examination only, surveillance was not associated with colorectal cancer incidence; however, in those patients with high-risk polyps only, or in those with both risk factors, surveillance was associated with lower cancer incidence (table 3).

**Figure 2:**
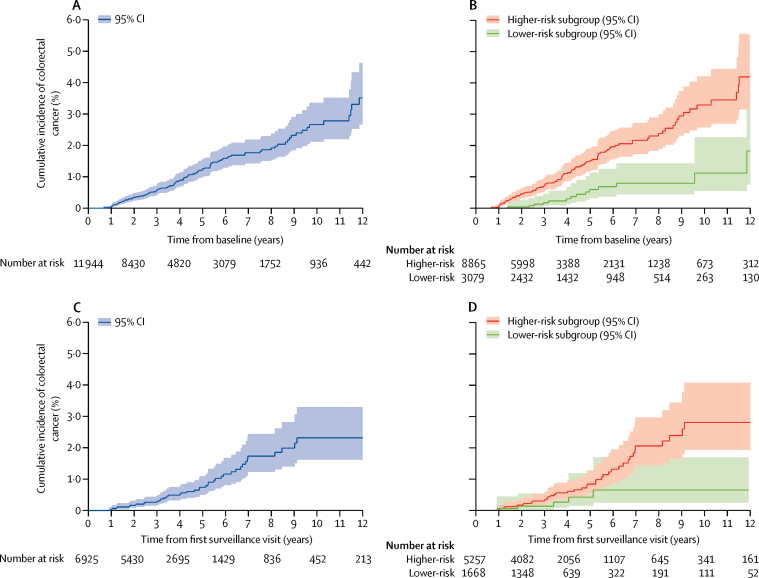
Cumulative colorectal cancer incidence after baseline Cumulative colorectal cancer incidence with no surveillance (ie, censoring at first follow-up) for the whole cohort (A) and for the risk subgroups (B). Cumulative colorectal cancer incidence after one surveillance visit (ie, censoring at the second follow-up) for the whole cohort (C) and for the risk subgroups (D). 95% CIs are shown for each curve.

Without surveillance, colorectal cancer incidence at 10 years was 2·7% (95% CI 2·1–3·4; 114 cancers) in the cohort overall; incidence was 3·3% (2·6–4·2; 101 cancers) in the higher-risk subgroup and 1·1% (0·5–2·3; 13 cancers) in the lower-risk subgroup (table 4). Colorectal cancer incidence in the whole cohort was not significantly different from that of the general population (SIR 1·09, 95% CI 0·91–1·30); however, colorectal cancer incidence was significantly higher in the higher-risk subgroup (SIR 1·30, 1·06–1·57) and significantly lower in the lower-risk subgroup (SIR 0·51, 0·29–0·84) than in the general population (table 4; figure 2A and B).

**Table 4:** Cumulative colorectal cancer incidence and age-and-sex standardised incidence ratios

		**n (%)**	**Person-years**	**Colorectal cancer cases**	**Incidence per 100 000 person-years (95% CI)**	**Follow-up**						**p value**	**Standardisation**	
						3 years		5 years		10 years			Number of colorectal cancers expected†	Standardised incidence ratio (95%CI)
						Colorectal cancer cases	Cumulative incidence (95% CI)*	Colorectal cancer cases	Cumulative incidence (95% CI)*	Colorectal cancer cases	Cumulative incidence (95% CI)*			
After baseline (no surveillance, censored at first surveillance)		..	..	..	..	..	..	..	..	..	..	<0·0001	..	..
	Whole cohort	11 944 (100%)	51 942	121	233 (195–278)	50	0·6% (0·4–0·7)	84	1·3% (1·0–1·6)	114	2·7% (2·1–3·4)	..	111	1·09 (0·91–1·30)
	Lower-risk subgroup‡	3079 (26%)	14 861	15	101 (61–167)	4	0·2% (0·06–0·5)	10	0·6% (0·3–1·1)	13	1·1% (0·5–2·3)	..	29	0·51 (0·29–0·84)
	Higher-risk subgroup‡	8865 (74%)	37 081	106	286 (236–346)	46	0·7% (0·5–1·0)	74	1·5% (1·2–1·9)	101	3·3% (2·6–4·2)	..	82	1·30 (1·06–1·57)
After first surveillance (one surveillance visit only, censored at second surveillance)		..	..	..	..	..	..	..	..	..	..	0·0431	..	..
	Whole cohort	6925 (100%)	29 503	51	173 (131–227)	16	0·3% (0·2–0·5)	29	0·7% (0·5–1·1)	47	2·3% (1·6–3·3)	..	64	0·80 (0·59–1·05)
	Lower-risk subgroup‡	1668 (24%)	7095	6	85 (38–188)	2	0·1% (0·03–0·5)	4	0·4% (0·1–1·2)	5	0·7% (0·2–1·7)	..	14	0·42 (0·16–0·92)
	Higher-risk subgroup‡	5257 (76%)	22 408	45	201 (150–269)	14	0·3% (0·2–0·6)	25	0·8% (0·5–1·3)	42	2·8% (1·9–4·1)	..	50	0·90 (0·66–1·21)
After second surveillance (two or more surveillance visits, censored at end of follow-up)		..	..	..	..	..	..	..	..	..	..	0·0991	..	..
	Whole cohort	3422 (100%)	19 589	38	194 (141–267)	10	0·4% (0·2–0·7)	14	0·6% (0·3–1·0)	29	2·0% (1·4–3·1)	..	44	0·86 (0·60–1·17)
	Lower-risk subgroup‡	731 (21%)	3749	3	80 (26–248)	1	0·2% (0·03–1·5)	2	0·5% (0·1–1·9)	3	1·3 (0·3–4·9)	..	8	0·36 (0·07–1·06)
	Higher-risk subgroup‡	2691 (79%)	15 840	35	221 (159–308)	9	0·4% (0·2–0·8)	12	0·6% (0·3–1·1)	26	2·2 (1·5–3·4)	..	36	0·97 (0·67–1·34)

p values are for the log-rank test comparing incidence in the higher-risk versus the lower-risk subgroup.

* One minus the Kaplan-Meier estimator of the survival function was used to estimate the cumulative incidence of colorectal cancer.

† Expected colorectal cancers were calculated by multiplying the sex and 5-year age-group-specific incidence rates in the general population of England in 2007 by the age-and-sex specific numbers of observed person-years.

‡ The higher-risk subgroup included patients with any of the following risk factors at baseline: suboptimal quality examination (defined as incomplete colonoscopy, unknown completeness, or poor bowel preparation), high-risk polyps (defined as proximal polyps or a high-grade or large [20mm or larger] adenoma), or both; the lower-risk subgroup included patients without any of these risk factors.

After a single surveillance visit, colorectal cancer incidence at 10 years was 2·3% (95% CI 1·6–3·3; 47 cancers) in the cohort overall, 2·8% (1·9–4·1; 42 cancers) in the higher-risk subgroup, and 0·7% (0·2–1·7; five cancers) in the lower-risk subgroup (table 4). Compared with the general population, the SIR for colorectal cancer was 0·80 (95% CI 0·59–1·05) in the overall cohort, 0·42 (95% CI 0·16–0·92) in the lower-risk subgroup, and 0·90 (95% CI 0·66–1·21) in the higher-risk subgroup (table 4; figure 2C and D). Following a second surveillance visit, colorectal cancer incidence at 10 years was 2·0% (1·4–3·1; 29 cancers) overall and 2·2% (1·5–3·4; 26 cancers) in the higher-risk subgroup; the lower-risk subgroup analyses were underpowered because only three colorectal cancers had been diagnosed by 10 years (table 4).

## Discussion

In this retrospective, multicentre, cohort study, colonoscopy surveillance was associated with a substantially reduced incidence of colorectal cancer in these intermediate-risk patients, who are currently offered surveillance colonoscopy at 3-year intervals. The first surveillance visit seemed to confer the most benefit and was associated with a significantly reduced colorectal cancer incidence rate compared with no surveillance; this incidence reduction was maintained in patients who attended subsequent visits. In the UK, about 20% of colonoscopies are done for the purpose of surveillance.^21^ In our dataset 80% of patients undergoing adenoma surveillance were at intermediate risk (figure 1).^21^

We identified a subgroup of patients at higher risk of colorectal cancer, which included roughly three-quarters of this intermediate-risk cohort. This subgroup consisted of patients who had a suboptimal quality colonoscopy (incomplete, of unknown completeness, or poor bowel preparation), a large adenoma (≥20 mm), an adenoma with high-grade dysplasia, or proximal polyps detected at baseline; surveillance was highly effective in this subgroup and was associated with a significant reduction in the incidence of colorectal cancer. By contrast, in patients without these baseline findings, the benefit of surveillance was unclear because only a few cancers were subsequently diagnosed.

Patients with intermediate-risk adenoma are offered surveillance at 3-year intervals because they are perceived to be at increased risk of colorectal cancer compared with the general population. This perception is based on high detection rates of advanced neoplasia in those who attend surveillance^11^, ^12^, ^13^, ^14^ and on follow-up of patients diagnosed in the 1980s and 1990s;^12^, ^19^, ^20^ colorectal cancer risk has not previously been quantified by use of data in an era of higher quality colonoscopies. We found that colorectal cancer incidence in the absence of surveillance was similar to that expected in the general population, suggesting that intensive surveillance might not be appropriate for all intermediate-risk patients. However, in the higher-risk subgroup, colorectal cancer incidence without surveillance was significantly higher than that of the general population; therefore, individuals in this subgroup might benefit from at least one surveillance visit. By contrast, in the lower-risk subgroup, the colorectal cancer incidence was already lower than that of the general population after baseline colonoscopy, with a 10-year cumulative incidence of only 1·1%. This low baseline incidence raises uncertainty as to whether any surveillance is warranted for these individuals.

Some of the independent risk factors for colorectal cancer that we identified within this intermediate-risk group have been described as risk factors for detection of advanced neoplasia at follow-up colonoscopy, including larger adenoma size, older patient age, and having only a suboptimal quality baseline colonoscopy.^11^, ^12^, ^13^, ^14^, ^15^, ^16^ A less well documented risk factor was the presence of polyps in the proximal colon, which in our study was associated with an increased incidence of colorectal cancer. This finding corroborates data from two previous studies reporting that patients with proximal polyps had an 80% increased risk of advanced neoplasia at follow-up colonoscopy.^14^, ^32^ This evidence suggests that proximal polyps could be regarded as a colorectal cancer risk factor in future iterations of surveillance guidelines. Data on lifestyle risk factors were not available. However, results from a large pooled analysis of 9167 men and women showed that body-mass index, smoking, and family history, which are often important epidemiological risk factors, are not major predictors of metachronous advanced neoplasia at surveillance after adjustment for the baseline adenoma characteristics.^14^

Our results emphasise the importance of achieving a complete colonoscopy with good quality bowel preparation. Having a suboptimal quality baseline examination was associated with a doubling in colorectal cancer incidence irrespective of polyp characteristics. In the UK, colonoscopies are done by gastroenterologists, surgeons, and specialist nurses. Since the national colonoscopy audit in 2001,^33^ there has been heightened awareness of colonoscopy standards and implementation of national quality assessments and training programmes,^21^, ^34^, ^35^ resulting in substantial improvements in endoscopy quality and leading to nearly 30% fewer cancers arising from missed or incompletely removed lesions within 3 years of colonoscopy in 2007 than in 2001.^36^

Patient factors, such as older age, female sex, having prior abdominal or pelvic surgery, and obesity might also affect the quality of a bowel preparation or colonoscopy.^37^, ^38^, ^39^, ^40^, ^41^, ^42^ In the English Bowel Cancer Screening Programme (BCSP), examinations to complete an investigation of the colon and remove detected lesions are regarded as part of the initial work-up, with surveillance only considered when baseline examinations have been completed; this would be a good policy to adopt for patients diagnosed with adenomas outside of the BCSP. For individuals in whom colonoscopy is problematic, the clinician should establish on a case-by-case basis whether it is appropriate to recommend colonoscopy surveillance.

In our study, 42% of patients did not attend surveillance. More non-attenders than attenders were female, aged 75 years or older, or had an incomplete colonoscopy or poor bowel preparation. Other factors that we were unable to assess but which are likely to affect attendance for surveillance include the health status of the patient, administrative problems in scheduling an appointment 3 years in advance, and patient choice, especially if they had either a bad experience with the index colonoscopy or the reasons for surveillance were not well explained.

The main strengths of this study are the generation of a high-quality detailed dataset by use of a large nationwide sample of routinely collected clinical endoscopy and pathology data on colonoscopies for consecutive patients with adenomas across 17 UK hospitals, which serve a combined population of more than 6·5 million people.^23^ Follow-up for cancer and death was complete for almost all patients and, apart from data on bowel preparation quality, very few data were missing. Finally, we studied incidence in a large number of patients with intermediate-risk adenomas, about 84% of whom had their baseline colonoscopy after the implementation of national quality improvement programmes beginning in 2000.

The main limitation of this study is that it is an observational study and therefore we cannot assume a causal association between surveillance and colorectal cancer incidence. However, we saw a large significant effect of surveillance both before and after adjustment for several potential confounding factors. Standardised data cleaning further minimised the risk of bias arising from measurement error or misclassification, although some misclassification is inevitable within routinely collected data. However, this misclassification is likely to have been non-differential and would have been more likely to have caused an underestimation of effects. Missing values were more common in patients attending surveillance than in those who did not. This difference was only substantial for the bowel preparation and colonoscopy completeness variables, suggesting that when a future surveillance visit was planned, there was less of a tendency to record the quality of the initial examination. A further limitation is that conclusions were based on a median of 7·9 years of follow-up and longer-term follow-up is needed to substantiate our findings, especially in the lower-risk subgroup without surveillance. Finally, although follow-up examinations were assumed to be for surveillance, some might have been for symptomatic purposes.

We conclude from our results that patients diagnosed with intermediate-risk adenomas are at only a small increased risk of developing colorectal cancer after their baseline colonoscopy and polypectomy compared with the general population, especially if they have had a good quality baseline colonoscopy; therefore, it is unclear whether all of these intermediate-risk patients need the currently recommended 3-yearly surveillance by colonoscopy. Among patients in the lower-risk subgroup, surveillance might not be warranted at all if baseline colonoscopy is complete, with good visibility of the bowel mucosa and all lesions completely excised. Patients with a suboptimal quality examination at baseline should have a good quality colonoscopy before their surveillance strategy is determined. In patients for whom a good quality colonoscopy is not possible, an alternative form of surveillance should be sought if appropriate. Patients with large adenomas (≥20 mm), high-grade dysplasia, or proximal polyps are likely to benefit significantly from at least one surveillance examination. Future studies should examine whether alternative strategies to surveillance colonoscopy might suffice for some patients. Additionally, future research should aim to define the subgroup of intermediate-risk patients for whom the risk of colorectal cancer after first surveillance is so low that they can stop surveillance altogether.
