# Supplementary material for: Adenoma surveillance and colorectal cancer incidence: a retrospective, multicentre, cohort study
Source: Lancet Oncol. 2017 Jun;18(6):823–34. doi: 10.1016/S1470-2045(17)30187-0 (PMC5461371; doi:10.1016/S1470-2045(17)30187-0)
Supplement: Supplementary appendix [file mmc1.pdf]

THE LANCET
Oncology

Supplementary appendix

This appendix formed part of the original submission and has been peer reviewed. We post it as supplied by the authors.

Supplement to: Atkin W, Wooldrage K, Brenner A, et al. Adenoma surveillance and colorectal cancer incidence: a retrospective, multicentre, cohort study. *Lancet Oncol* 2017; published online April 27. http://dx.doi.org/10.1016/S1470-2045(17)30187-0.

SUPPLEMENTARY MATERIAL

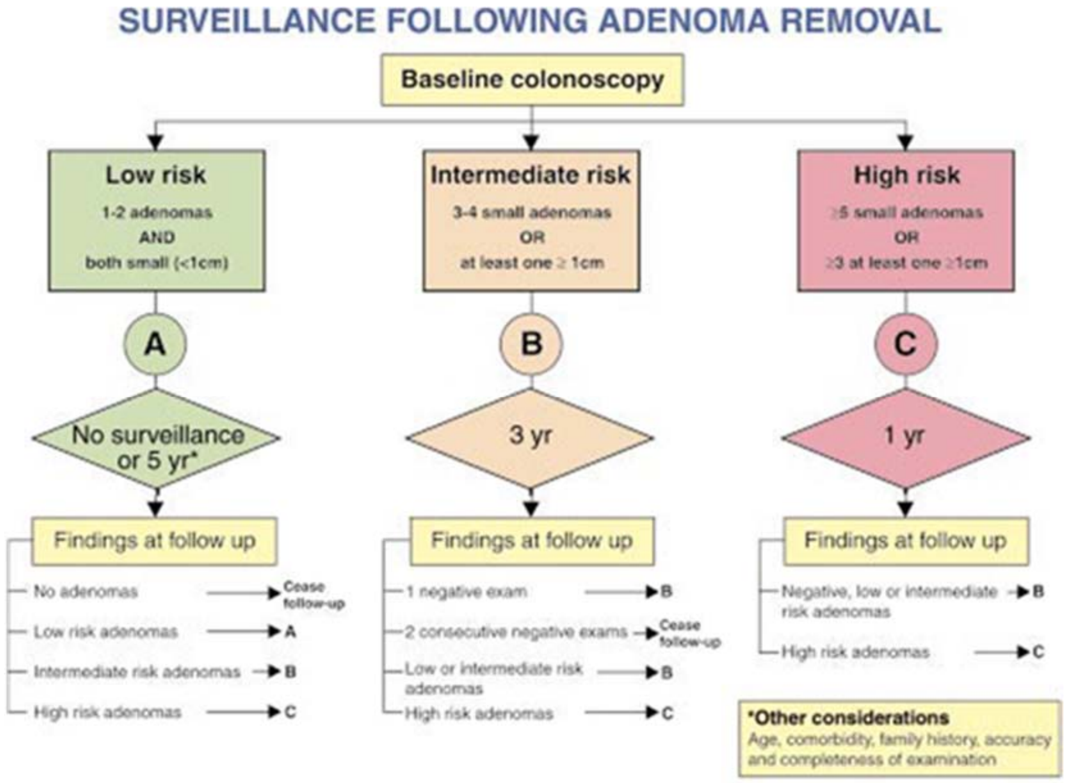

Supplementary Figure 1: UK adenoma surveillance guidelines 2002.[6]

Supplementary Table 1: Patient inclusion by hospital

Trust	Hospital	Principal Investigator	Number of patients included in study
Greater Glasgow and Clyde NHS Board	Glasgow Royal Infirmary	Mr. John Anderson	1,972
Royal Liverpool and Broadgreen University Hospitals NHS Trust	Royal Liverpool University Hospital	Dr Michael Burkitt/Prof Tony Morris	1,236
Imperial College Healthcare NHS Trust	Charing Cross Hospital/ Hammersmith Hospital/St Mary's Hospital	Mr Paul Ziprin/ Dr. Geoff Smith	1,218
London North West Healthcare NHS Trust	St Mark's Hospital	Prof Wendy Atkin	1,148
St George's University Hospitals NHS Foundation Trust	St George's Hospital	Mr. Roger Leicester	1,046
Royal Wolverhampton Hospitals NHS Trust	New Cross Hospital	Dr. Andrew Veitch	814
North Cumbria University Hospitals NHS Trust	Cumberland Infirmary	Dr. Denis Burke	654
Royal Surrey County Hospital NHS Trust	Royal Surrey County Hospital	Mr. John Stebbing	643
Shrewsbury and Telford Hospitals NHS Trust	Royal Shrewsbury Hospital	Dr. Mark Smith	608
University Hospitals of Leicester NHS Trust	Leicester General Hospital	Dr. John de Caestecker	566
Torbay and South Devon NHS Foundation Trust	Torbay District General Hospital	Dr. Mark Feeney	509
Yeovil District Hospital NHS Foundation Trust	Yeovil District Hospital	Mrs Sue Bulley	431
North Tees and Hartlepool Hospitals NHS Foundation Trust	University Hospital of North Tees	Prof Matt Rutter	426
Brighton & Sussex University Hospitals NHS Trust	Royal Sussex County Hospital	Dr. Stuart Cairns	252
Oxleas NHS Foundation Trust	Queen Mary's Hospital, Sidcup	Dr. Howard Curtis/Dr. Guy Sisson	241
Lewisham and Greenwich NHS Trust	Queen Elizabeth Hospital, Woolwich	Dr. Alistair McNair	180

Supplementary Table 2: Long-term colorectal cancer incidence by baseline patient, polyp and procedural factors not included in Table 2

	n (%)	Person-years	Colorectal cancer cases	Incidence per 100 000 person-years (95% CI)	Univariable hazard ratio (95% CI)	p value	Multivariable hazard ratio (95% CI)	p value
Total	11 994 (100%)	101 034	210	208 (182-238)				
Family history of cancer						0.39		0.57
No	11 445 (95.8%)	96 068	202	210 (183-241)	1 (Referent)		1	
Yes	499 (4.2%)	4 966	8	161 (81-322)	0.74 (0.37-1.51)		1.24 (0.60-2.54)	
Length of baseline visit						0.0553		0.1020
1 day	6 836 (57.2%)	57 943	99	171 (140-208)	1		1	
2-30 days	734 (6.1%)	5 601	16	286 (175-466)	1.73 (1.02-2.93)		1.61 (0.94-2.75)	
1-3 months	1 643 (13.8%)	14 068	36	256 (185-355)	1.52 (1.04-2.23)		1.55 (1.05-2.29)	
3-6 months	1 382 (11.6%)	11 953	26	218 (148-319)	1.29 (0.84-1.98)		1.20 (0.77-1.87)	
6-12 months	1 177 (9.9%)	10 123	29	286 (199-412)	1.68 (1.11-2.54)		1.65 (1.08-2.53)	
≥12 months	172 (1.4%)	1 346	4	297 (112-792)	1.77 (0.65-4.81)		1.60 (0.58-4.44)	
Number of hyperplastic polyps						0.84		0.86
0	9 874 (82.7%)	83 523	178	213 (184-247)	1		1	
1	1 307 (10.9%)	11 139	21	189 (123-289)	0.90 (0.57-1.41)		1.00 (0.63-1.58)	
2	405 (3.4%)	3 345	4	120 (45-319)	0.57 (0.21-1.53)		0.65 (0.24-1.75)	
3	152 (1.3%)	1 270	3	236 (76-732)	1.14 (0.36-3.57)		1.33 (0.42-4.17)	
4	76 (0.6%)	653	2	306 (77-1224)	1.45 (0.36-5.83)		1.99 (0.49-8.08)	
≥5	130 (1.1%)	1 104	2	181 (45-724)	0.86 (0.21-3.46)		1.17 (0.29-4.73)	
Any large hyperplastic polyps?						0.89		0.99
No	11 761 (98.5%)	99 455	207	208 (182-239)	1		1	
Yes	183 (1.5%)	1 579	3	190 (61-589)	0.92 (0.29-2.88)		0.99 (0.32-3.10)	
Number of polyps with unknown histology						0.36		0.16
0	9 322 (78.0%)	79 996	174	218 (187-252)	1		1	
1	1 510 (12.6%)	12 171	16	131 (81-215)	0.62 (0.37-1.03)		0.55 (0.33-0.93)	
2	517 (4.3%)	4 016	8	199 (100-398)	0.94 (0.46-1.92)		0.86 (0.42-1.77)	
3	249 (2.1%)	2 009	5	249 (104-598)	1.15 (0.47-2.81)		1.03 (0.42-2.55)	
4	129 (1.1%)	1 053	4	380 (143-1012)	1.77 (0.66-4.78)		1.85 (0.68-5.04)	
≥5	217 (1.8%)	1 788	3	168 (54-520)	0.77 (0.25-2.42)		0.63 (0.20-2.01)	

The reported multivariable hazard ratio and associated p value were for if the variable was added as an additional variable to the final multivariable model presented in Table 2 (the final model included number of surveillance visits, age, adenoma size, adenomas dysplasia, proximal polyps, completeness of colonoscopy and bowel preparation quality).

Supplementary Table 3: Number of surveillance visits, age and year of entry in the lower- and higher-risk subgroups

	n	Lower-risk subgroup n (%)	Higher-risk subgroup† n (%)	p value
Total	11 944	3 079 (25.8%)	8 865 (74.2)	
Number of surveillance visits after baseline				<0.0001
0	5 019	1 411 (45.8%)	3 608 (40.7%)	
1	3 503	937 (30.4%)	2 566 (29.0%)	
2	2 085	506 (16.4%)	1 579 (17.8%)	
≥3	1 337	225 (7.3%)	1 112 (12.5%)	
Age at first adenoma detection (years)				<0.0001
<55	2 122	643 (20.9%)	1 479 (16.7%)	
55-64	3 179	882 (28.6%)	2 297 (25.9%)	
65-74	3 957	1 001 (32.5%)	2 956 (33.3%)	
≥75	2 686	553 (18.0%)	2 133 (24.1%)	
Year of entry (start of baseline)				<0.0001
1984-1994	439	85 (2.8%)	354 (4.0%)	
1995-1999	1 430	317 (10.3%)	1 113 (12.6%)	
2000-2004	4 251	942 (30.6%)	3 309 (37.3%)	
2005-2010	5 824	1 735 (56.3%)	4 089 (46.1%)	

P values calculated with χ^2 test to compare patients in the lower- and higher-risk subgroups.
†The higher-risk subgroup included patients with any of the following risk factors at baseline: sub-optimal quality examination (defined as incomplete colonoscopy, unknown completeness, or poor bowel preparation), high risk polyps (defined as 20mm or larger adenoma or proximal polyps or having high grade dysplasia), or both.
